# Compulsive Eating in a Rat Model of Binge Eating Disorder Under Conditioned Fear and Exploration of Neural Mechanisms With *c-fos* mRNA Expression

**DOI:** 10.3389/fnins.2021.777572

**Published:** 2021-11-29

**Authors:** Zhi Fei Li, Sandrine Chometton, Geneviève Guèvremont, Elena Timofeeva, Igor Timofeev

**Affiliations:** ^1^The First Affiliated Hospital, Jinan University, Guangzhou, China; ^2^Faculté de Médecine, Département de Médecine Moléculaire, Université Laval, Quebec City, QC, Canada; ^3^Centre de Recherche de l’Institut Universitaire de Cardiologie et de Pneumologie de Québec, Quebec City, QC, Canada; ^4^Centre de Recherche CERVO, Quebec City, QC, Canada

**Keywords:** binge eating disorder, compulsive eating, reward devaluation, *c-fos* expression, fear conditioning, nucleus accumbens

## Abstract

Compulsive eating is the most obstinate feature of binge eating disorder. In this study, we observed the compulsive eating in our stress-induced binge-like eating rat model using a conflicting test, where sucrose and an aversively conditioned stimulus were presented at the same time. In this conflicting situation, the binge-like eating prone rats (BEPs), compared to the binge-like eating resistant rats (BERs), showed persistent high sucrose intake and inhibited fear response, respectively, indicating a deficit in palatability devaluation and stronger anxiolytic response to sucrose in the BEPs. We further analyzed the neuronal activation with *c-fos* mRNA *in situ* hybridization. Surprisingly, the sucrose access under conditioned fear did not inhibit the activity of amygdala; instead, it activated the central amygdala. In the BEPs, sucrose reduced the response of the paraventricular hypothalamic nucleus (PVN), while enhancing activities in the lateral hypothalamic area (LHA) to the CS. The resistance to devaluating the palatable food in the BEPs could be a result of persistent Acb response to sucrose intake and attenuated recruitment of the medial prefrontal cortex (mPFC). We interpret this finding as that the reward system of the BEPs overcame the homeostasis system and the stress-responding system.

## Introduction

Binge eating disorder (BED) is characterized by discrete episodes of overeating within a short period of time, usually less than 2 h, even when not feeling hungry (Bogduk, 2013). People with BED will continuously eat until physically feeling uncomfortable. In humans, negative consequences associated with overeating include social impairment, emotional disturbances, psychiatric disorders, and life-threatening medical conditions associated with weight gain (Moore et al., 2017).

Stress is one of the main inducers of BED in human patients. Evidence from both human and animal studies revealed that stress could have bidirectional influence on feeding behaviors, either inhibiting or stimulating food intake (Maniam and Morris, 2012). The direction of this changing effect of stress on feeding is dependent on the palatability of the food following stress (Pecoraro et al., 2004). A variety of rodent models of the BED have been developed by using food deprivation and/or stress. Our lab previously developed a binge-like eating rat model through intermittent foot shock stress followed by 1-h sucrose access (Calvez and Timofeeva, 2016). In this model, the binge-eating prone rats (BEPs) consume more sucrose solution than the binge-eating resistant rats (BERs) both in normal condition and after stress, and the BEPs further increase their sucrose intake after the stress stimulation.

Another hallmark of the BED is the compulsive eating, demonstrated as repetitively returning into engorging unhealthy food with full knowledge of the hazardous physical and psychological consequences. Both our BED rat model and the animal models stated in other literature noticed compulsive eating, in a modified Light/Dark box test (Calvez and Timofeeva, 2016) and unconditioned stimulus (US) such as foot shock (Oswald et al., 2011), respectively. The BEPs consumed larger amount of sucrose than the BERs in the intensively illuminated light box. In response to stress, the BEPs demonstrated a hyporeactivity of the hypothalamic–pituitary–adrenal (HPA) axis compared with the BERs (Calvez et al., 2016).

To gain a better understanding of the compulsive eating in the BED, we adopted the fear conditioning paradigm. Aversively conditioned stimuli have an inhibitory effect on the feeding behavior, and this effect can be abolished by lesions of the central nucleus but not the basolateral nucleus of the amygdala (Petrovich et al., 2009). Thus, the first objective of this study is to observe compulsive eating in our BED rat model by creating a conflicting situation with simultaneous presence of a 10% sucrose solution and an aversively conditioned stimulus. Based on that, we hypothesized that (1) the abnormally intense motivation for palatable food in the BEPs would attenuate the inhibitory effect of the CS on feeding, and (2) the BEPs would show less fear response to the CS because of a stronger anxiolytic effect of palatable food on the BEPs relative to BERs. The second objective is to explore the underlying neural mechanisms *via* analyzing the *c-fos* mRNA expression in different brain regions related to feeding, reward processing, and stress responding. The *c-fos* gene is one of the immediate early genes widely used as a marker of early neuronal activation (Kovács, 1998), because its expression is correlated with the functional activation of neurons. As a result, we hypothesized that different levels of *c-fos* mRNA expression in brain regions underlying food intake and stress response would be observed between the BEPs and BERs after fear conditioning test.

## Materials and Methods

### Animals

Young (PD 45, 151–175 g) female Sprague Dawley rats (*n* = 170) were purchased from Charles River. All rats were individually housed in transparent plastic cages, lined with wood shavings and crinkle paper. The rats were maintained on a 12-h light/dark cycle (lights on from 2:00 to 14:00), and provided with *ad libitum* access to standard laboratory rat chow (Teklad Global 18% Protein Rodent Diet; 3.1 kcal/g, Harlan Teklad, Montreal, QC, Canada) and tap water, unless noted otherwise. All rats were acclimated to the housing conditions and handling procedures for at least 1 week prior to the experiments.

### Classification of the Binge-Like Eating Prone Rats and Binge-Like Eating Resistant Rats

Rats were classified as the BERs or the BEPs according to the procedures previously described (Calvez and Timofeeva, 2016). All rats were given a 24-h access to a 10% sucrose solution in their home cages to decrease their neophobia to sucrose. Then, we assessed the consumption of 10% sucrose solution (0.4 kcal/ml) during several intermittent 1-h sessions starting from the beginning of the dark phase in home cages, with random intertrial intervals (ITIs) of 1 or 2 days. When the sucrose intake became stable for three consecutive No Stress sessions, three Stress sessions were conducted with intervals of 2 or 3 days. In the Stress sessions, 1-h sucrose access was provided in home cages immediately after four rounds of mild foot shock in a procedure room (0.6 mA DC impulse, 3 s duration, with inter-shock intervals of 15 s). The second and third Stress sessions were separated by a No Stress session to prevent the rats from associating the sucrose access with foot shock. The food pellets were removed during sucrose access in home cages, and put back immediately after each session ended. Sucrose intake of all animals in each Stress session was divided into high, intermediate, and low intake tertiles. Rats with sucrose intake in the high tertiles at least twice and never in the low tertiles were sorted as binge-like eating prone, while rats with sucrose intake in the low tertiles at least twice and never in the high tertiles were classified as binge-like eating resistant. After the phenotyping, 42 rats were categorized into the BEP group and 44 rats into the BER group. In order to keep this animal model consistent with our previously published studies, sucrose intake without normalization by body weight was used for the phenotyping.

### The Fear Conditioning Test

**FIGURE 1:**
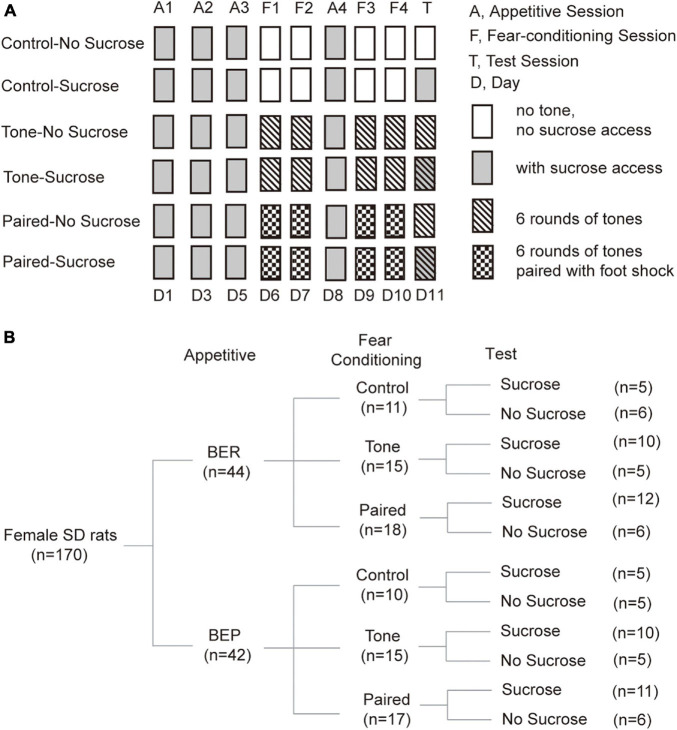
Diagram of treatments and groups in the Fear Conditioning test. **(A)** Diagram of fear conditioning test procedures. **(B)** Number of animals in each group at each step during the fear conditioning test.

The fear conditioning test was composed of three parts: Habituation and Appetitive sessions, Fear Conditioning sessions, and the Test session (Figure 1A). All sessions lasted for 15 min, and were videotaped (Logitech HD Webcam C270) from the top of the sound-attenuating cubicle (Med Associates inc.; ENV-022V, 55.9 cm × 38.1 cm × 35.6 cm) containing the behavioral test chamber (Med Associates inc.; ENV-007-VP, 30.5 cm × 24.1 cm × 29.2 cm). The test chamber had a grid floor and aluminum sides, and there were Plexiglas in the front and the top sides, except for the back side. The test chamber was illuminated with a light (4 W) placed 25 cm above the floor. A speaker (Med Associates inc.; ENV-224AM) was installed on the left wall, 20 cm above the floor. Two photobeam lickometers (Med Associates inc.; ENV-251L) were installed on the front and back parts of the right wall, 3 cm above the floor, supplied with a bottle of 10% sucrose solution and water, respectively. A door in front of the sucrose lickometer was controlled by a custom-designed program (Med-PC V, Med Associates inc.) to start and end the sucrose access.

#### Habituation and Appetitive Sessions

For the Habituation and Appetitive sessions, the test chamber was arranged as context A. In this context, a white plastic panel was installed on the grid floor and apple-scented beads were placed in the sound attenuating box. The light was placed on the top right corner of the left wall. Furthermore, the test chamber was cleaned with Percept cleaner between each rat, and the animals were transported from the housing room to the test room on a cart. In this context, the rats were habituated to drink 10% sucrose solution in the behavioral test chamber with ITIs of 1 or 2 days at random, until the sucrose intake became stable for three sessions. Thus, the last three sessions during which the sucrose intake of BER and BEP groups showed no significant changes were defined as Appetitive sessions (Figure 1A).

#### Fear Conditioning Sessions

In Fear Conditioning sessions, the behavioral cage was modified into context B to be different from the context of Appetitive sessions. The plastic floor and apple-scented beads were removed. The light was moved to the top right corner of the right wall. A piece of curved white plastic board was placed to serve as the back and sidewalls of the test chamber and there was neither sucrose nor water access for the rats. The test chamber was cleaned with Citrosol cleaner between each rat and the BEPs and BERs were transported by the experimenter. In this stage, we divided rats of both phenotypes into three groups (Control, Tone, and Paired groups; Figure 1B).

(a) For the Paired groups, a mild foot shock (1 s, 0.6 mA, DC impulses) was delivered through the grid floor during the last second of each of the six tones (the first tone started 20 s after the onset of the trial, 20 s, 2 kHz, 75 dB), separated by 125-s no-stimulus periods.
(b) For the Tone groups, the same condition was applied, but no foot shock was delivered in these sessions.
(c) For the Control groups, no tone or foot shock was applied during each 15-min Fear Conditioning session.

Between the second and third Fear Conditioning sessions, an Appetitive session was inserted to prevent the rats from losing their phenotype (Figure 1A). In this Appetitive session, the behavioral cage was arranged as context A.

#### The Test Session

The test cage was arranged as context A in the Test session. During the Test session, context A was used as in the Appetitive session. We divided rats of each group in the Fear Conditioning sessions into two groups (No Sucrose and Sucrose; Figure 1B). Sucrose groups had access to 10% sucrose solution throughout the Test session, while the No Sucrose groups did not. For the Tone and Paired groups, six rounds of the same tones as in Fear Conditioning sessions were presented, but without foot shock stimulus (Figure 1A).

During the Appetitive sessions and the Test session, sucrose intake was assessed by weighing the bottle before and after the experiment, and the licking events were detected by the lickometer and recorded with a multichannel system (Tucker-Davis Technologies) and the Med Associates system (Med Associates inc.). Freezing behavior of the Paired groups was observed on video and quantified by the experimenter during the tones in Fear Conditioning sessions and the Test session.

### Licking Microstructure Analyses

The licking events were analyzed in all 12 groups (Figure 1B). In No Sucrose groups, the licks were too scarce for licking microstructure analysis and further statistical analysis, which was the reason that these licking misstructure results were absent for these groups in the Test session. The first lick latency was defined as the time in seconds between the beginning of the session, and the first lick of sucrose solution. A licking cluster was defined as a burst of three or more licks with inter-cluster intervals of 500 ms or longer. The meal duration was calculated as the total duration in seconds of all clusters during the 15-min access to sucrose solution. The cluster size was defined as the average number of licks per cluster, and the cluster duration was defined as the average duration of all clusters in seconds. The total number of licks, meal duration, number of clusters, cluster size, cluster duration, and first lick latency were computed for each session with a customized MATLAB script (R2014a, The MathWorks, inc.).

### Brain Preparation

Immediately after the Test session, each rat was returned to its home cage, with access to neither chow nor water for 30 min. Then, the rats were anesthetized with a mixture of ketamine (60 mg/kg) and xylazine (7.5 mg/kg) and intracardially perfused with ice-cold saline followed by 4% paraformaldehyde (PFA) in phosphate buffer. The brains were removed from the skulls and fixed in 4% PFA for 1 week before being transferred to a PFA (4%)/sucrose (20%) solution. After freezing, brains were conserved in −80°C. Each brain was cut into 30-μm coronal sections using a microtome (Histoslide 2000, Reichert Jung, Heidelberger, Germany). All sections from each brain were distributed into a 24-well plate filled with a cold sterile cryoprotecting solution containing ethylene glycol (30%), glycerol (20%), and sodium phosphate buffer (50 mM, pH 7.2), and stored at −30°C.

### *In situ* Hybridization for *c-fos* mRNA

The protocol of *in situ* hybridization we used to localize the *c-fos* mRNA in this study was largely adapted from the method described by Simmons et al. (1989). The procedures have been described in detail previously (Poulin and Timofeeva, 2008). Briefly, brain sections were mounted onto poly-L-lysine-coated slides and conserved in 100% ethanol. After the slides dried up, they were successively fixed in 4% PFA for 20 min, digested with proteinase K (0.01 mg/ml) at 37°C for 25 min, acetylated with acetic anhydride (0.25% in 0.1 M triethanolamine, pH 8.0), and dehydrated through ethanol gradient (50, 70, 95, and 100%). After the slides dried up, 90 μl of the hybridization solution containing a ^35^S labeled antisense cRNA probe against *c-fos* mRNA (De Ávila et al., 2018) was spread on each slide. All slides were then covered with coverslips and incubated overnight in a slide warmer at 60°C. After the coverslips were removed, the slides were rinsed four times with 4 × saline sodium citrate buffer (SSC, 0.6 M NaCl, 60 mM trisodium citrate buffer, pH 7.0), digested with RNase-A (20 μg/ml in 10 mM Tris–500 mM NaCl containing 1 mM EDTA) for 30 min at 37°C, rinsed in SSC with descending concentrations (2×, 1×, 0.5×, and 0.1×), and finally dehydrated through ethanol gradient. Thereafter, the slides were defatted in toluene, dipped in nuclear emulsion (Kodak), and exposed for 7 days before being developed in the developer (Kodak) and fixed in rapid fixer (Kodak). Finally, slides were rinsed in running cold tap water for 1 h, stained with thionin, dehydrated through an ethanol gradient, cleared in toluene, and coverslipped with DPX.

### Relative *c-fos* mRNA Expression Analyses

The slides were analyzed under a light microscope (Olympus) equipped with a camera coupled to a computer with Stereo Investigator software (v1103). The luminosity of the system was set to the maximum. To avoid saturation, the exposure time for each region was adjusted with the section that had the strongest hybridization signal. For every area of interest, two photos were taken under both bright-field and dark-field illumination without moving the slides at a magnification of 4×. Thus, the position of the area of interest on both images are exactly the same. When the borders of the area of interest were not clear on the dark-field images, the light-field images were used to assist drawing the contours on the dark-field image.

There are many brain regions related to BED. In this study, we want to explore the neural mechanism of compulsive property of binge eating. For this purpose, we created a conflicting situation and allowed the stress-induced binge-like eating rats to make the decision between responding to stress or palatable food. Thus, we examined the *c-fos* mRNA expression in brain regions closely related to stress responding, food intake regulation, and decision-making, namely, the amygdala (2.16–3.48 mm caudal to the bregma), the paraventricular hypothalamic nucleus (PVN, 1.56–1.80 mm caudal to the bregma), the lateral hypothalamic area (LHA, 2.28–3.48 mm caudal to the bregma), the nucleus accumbens (Acb, 2.28–1.08 mm rostral to the bregma), the bed nucleus of the stria terminalis (BNST, 0.36 mm rostral to 0.36 mm caudal to the bregma), and the medial prefrontal cortex (mPFC, 3.72–2.52 mm rostral to the bregma).

The area of interest was outlined on each photo by the experimenter with Stereo Investigator. The hybridization signal was quantified by calculating the optical density (OD) in the contour on the dark-field image with a customized MATLAB script (R2014a, The MathWorks, inc.). The OD of each area of interest was corrected by subtracting the background signal, which was determined by three small contours on the unlabeled areas around the area of interest.

Brain slices from different brain regions were labeled in different batches of *in situ* hybridization. Moreover, the exposure parameters were constant only for slices from the same brain region, while different from region to region. Thus, the optical densities were comparable only between images from the same brain region, but not between different brain regions.

### Statistical Analyses

Results are presented as mean ± standard deviation (SD). The phenotype effects on first lick latency in Appetitive sessions and the Test session were analyzed using one-way ANOVA. Two-way ANOVA with Bonferroni *post hoc* test was used for all other statistical analyses. A difference was considered significant when *p*-values < 0.05. Statistical analyses were performed using the Prism 6.04 (GraphPad Software inc., La Jolla, CA, United States), and graphs were made with Prism 6.04 and arranged into figures with Adobe Illustrator^®^ CS.

## Results

### Classification of the Binge-Like Eating Resistant Rats and Binge-Like Eating Prone Rats

The difference in sucrose intake between the BEPs and BERs during the phenotyping in our study was similar to previously published results (Calvez and Timofeeva, 2016). The BEPs had significantly higher sucrose intake than the BERs both in the non-stressful situation (*p* < 0.0001) and after foot shock stress (*p* < 0.0001). Moreover, the BEPs consumed even more sucrose after stress (*p* < 0.0001), but the stress showed no significant impact on the sucrose intake of the BERs (*p* = 0.998). Two-way ANOVA assessed the effect of stress (*F*_1_,_168_ = 24.530, *p* < 0.0001), phenotype (*F*_1_,_168_ = 168.400, *p* < 0.0001), and their interaction (*F*_1_,_168_ = 25.220, *p* < 0.0001) on the 1-h sucrose intake in both non-stressful and after stress situations (data not shown).

### Sucrose Intake Behavior During the Appetitive and Test Sessions

**FIGURE 2:**
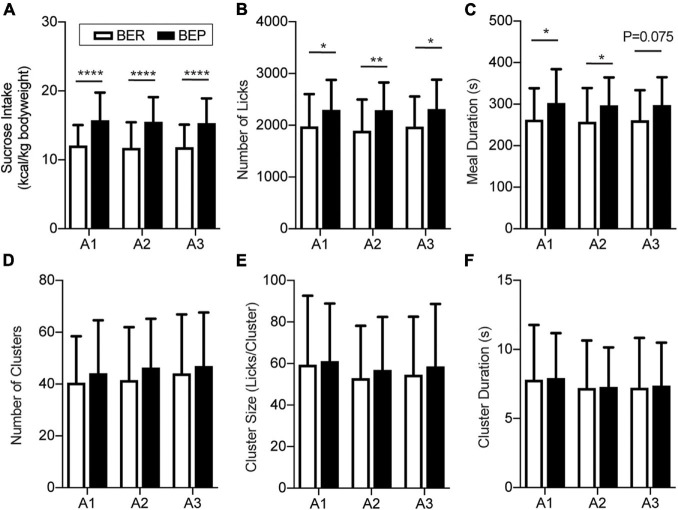
Feeding behavior during appetitive sessions. Binge-like eating prone (BEP) rats showed constant higher sucrose intake during the Appetitive sessions compared to binge-like eating resistant (BER) rats. **(A)** Sucrose intake normalized to body weight. Licking microstructures during the Appetitive sessions: total number of licks **(B)**, meal duration **(C)**, number of clusters **(D)**, cluster size **(E)**, and cluster duration **(F)**. *Significantly different (*p* < 0.05) between the BEP group and BER group. **p < 0.01, ****p < 0.0001.

There was no significant difference in the body weight between BER and BEP rats throughout the fear conditioning test (data not shown). Anyway, to eliminate the potential influence of body weight on the sucrose intake, the quantity of sucrose intake during Appetitive and Test sessions of each rat was calculated as the energy of the consumed sucrose normalized by its body weight (kcal/kg body weight). As the BEPs and BERs were accommodated in the behavioral test chamber, the 15-min sucrose intake of both phenotypes gradually reached a plateau. The last three sessions with stable sucrose consumption of the BEPs and BERs were defined as the Appetitive sessions (Figure 2A). Two-way ANOVA revealed a significant effect of phenotype on the sucrose intake in the Appetitive sessions. The BEPs took more sucrose than the BERs in all Appetitive sessions, which is consistent with their phenotypes in the home cage during the binge eating classification (Figure 2A). Analysis of the microstructure of licking events showed that the total number of licks was significantly higher in the BEPs than the BERs for the three Appetitive sessions (Figure 2B). The meal duration was also significantly higher in the BEPs than the BERs in the first and second sessions, and close to the significance in the third (Figure 2C). The number of clusters (Figure 2D), cluster size (Figure 2E), and cluster duration (Figure 2F) were not significantly different between the BEPs and BERs. Finally, the BEPs showed significantly lower average first lick latency in three Appetitive sessions than the BERs rats (two-tailed unpaired *t*-test, *p* = 0.003, data not shown).

**FIGURE 3:**
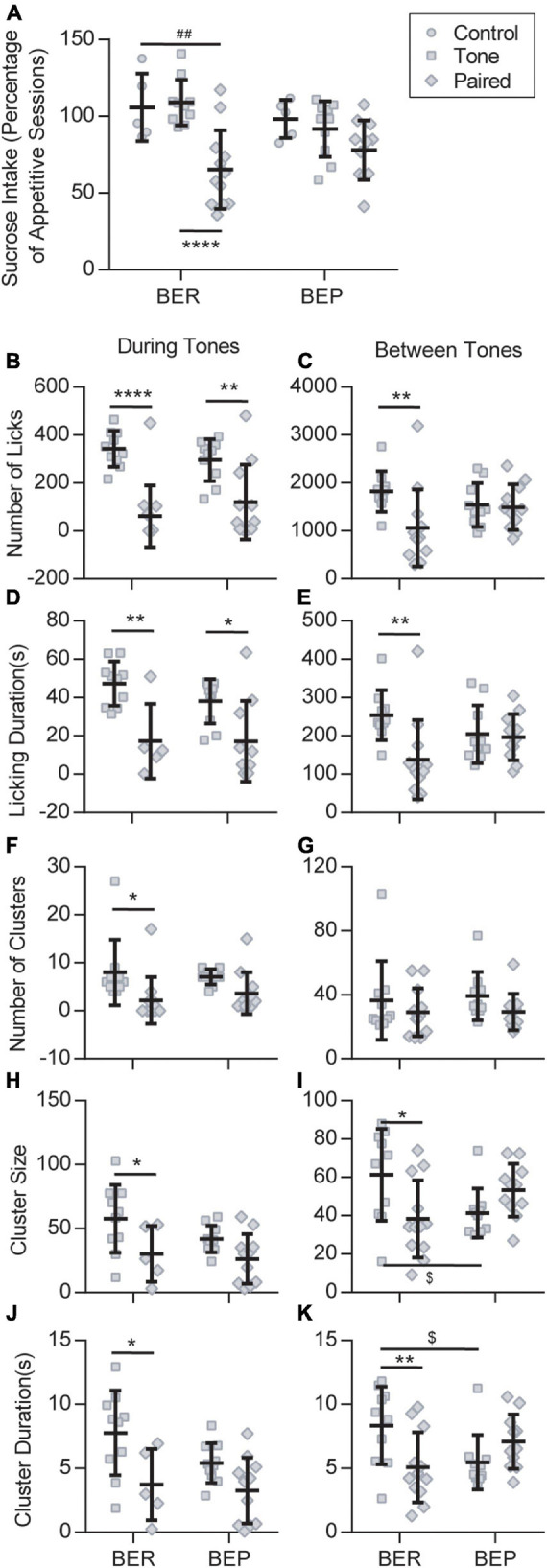
Feeding behavior during the Test session. **(A)** Sucrose intake during the Test session normalized to the average intake of each animal during all appetitive sessions. ^##^Significantly different (*p* < 0.01) from the BER-Control group. ****Significantly different (*p* < 0.0001) from the BER-Tone group. Licking microstructures during the Test session: number of licks **(B,C)**, licking duration **(D,E)**, number of clusters **(F,G)**, cluster size **(H,I)**, and cluster duration **(J,K)**. *Significantly different (*p* < 0.05) from the Tone group with the same phenotype. **p < 0.01, ****p < 0.0001. ^$^Significantly different (*p* < 0.05) from the BER-Tone group.

In the Test session, because of the scarcity of licks in the no-sucrose groups, we only considered the sucrose intake of the groups with sucrose access. To diminish the individual differences, we normalized the sucrose intake/body weight of each rat to its average sucrose intake/body weight in the Appetitive sessions. Two-way ANOVA revealed a significant effect of stress (*F*_2_,_47_ = 14.180, *p* < 0.0001) on the sucrose intake in the Test session, and a close to significant effect of its interaction with phenotype (*F*_2_,_47_ = 3.117, *p* = 0.054) but not of the phenotype itself (*F*_1_,_47_ = 0.481, *p* = 0.491). The result demonstrated that the unconditioned tones failed to change the sucrose intake of either the BEPs or the BERs (Figure 3A). When the tones were previously associated with foot shocks, they prominently decreased the sucrose intake of the BER-Paired-Sucrose group compared with the BER-Control-Sucrose group and the BER-Tone*-*Sucrose group (Figure 3A). The aversive CS slightly decreased the sucrose intake of BEPs, but without reaching a significant level (Figure 3A). We also analyzed the first lick latency in the Test session showing that the conditioned fear significantly increased the first lick latency of BER-Paired rats (*n* = 18) compared with the BEP-Paired rats (*n* = 17) (two-tailed unpaired *t-*test, *p* = 0.047, data not shown).

Next, we analyzed the licking microstructures during tones and between tones separately, in Tone and Paired groups. During tones, two-way ANOVA revealed a substantial effect of the CS on all licking microstructures, namely, the number of licks, licking duration (*F*_1_,_31_ = 20.280, *p* < 0.001), number of clusters (*F*_1_,_39_ = 0.038, *p* = 0.003), cluster size, and cluster duration, without the effect of phenotype or their interaction. Between tones, the CS only showed a significant effect on the number of licks and licking duration, while the interaction between CS and phenotype displayed a major influence on the number of licks, licking duration, cluster size, and cluster duration. For the BERs, both during tones and between tones, the conditioned fear significantly decreased the number of licks (Figures 3B,C), licking duration (Figures 3D,E), cluster size (Figures 3H,I), and cluster duration (Figures 3J,K). The number of clusters only decreased during tones (Figures 3F,G). In the BEPs, the conditioned fear only decreased the number of licks (Figure 3B) and the licking duration (Figure 3D) during tones, and the other analyzed licking microstructures were not significantly changed by the conditioned fear.

### Freezing Behavior During Fear Conditioning Sessions and the Test Session

**FIGURE 4:**
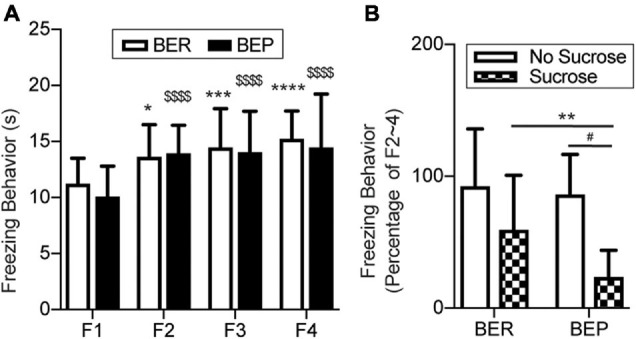
Conditioned fear response in training sessions and the test session. **(A)** Freezing behavior during the Training sessions. *Significantly different (*p* < 0.0.5) from the F1 session within the BER group. ***p < 0.001; ****p < 0.0001. ^$$$$^Significantly different (*p* < 0.0001) from the F1 session within the BEP group. **(B)** Freezing behavior during the Training sessions normalized to the average freezing behavior of each animal during F2–4. F1–4, Fear Conditioning sessions 1–4. **Significantly (*p* < 0.05) different from the BER-Sucrose group. ^#^Significantly (*p* < 0.01) different from the BEP-No Sucrose group.

The fear response of each rat was measured as the average freezing time during six tones in each session. In the Fear Conditioning sessions, the BER-Paired and BEP-Paired groups had similar acquisition efficiency and showed no phenotype difference throughout all four sessions (Figure 4A). The freezing time significantly increased in the second Fear Conditioning session compared with the first and became relatively stable afterward. Two-way ANOVA revealed a significant effect of the session number (*F*_3_,_99_ = 23.750, *p* < 0.0001) on the freezing behavior, but not the phenotype (*F*_1_,_33_ = 0.365, *p* = 0.550) or their interaction (*F*_3_,_99_ = 0.659, *p* = 0.579).

Again, to diminish individual differences, we normalized the freezing time of each rat in the Test session to its average freezing time of the last three Fear Conditioning sessions (Figure 4B). With the absence of foot shock during the Test session, the BER-Paired*-*No Sucrose and BEP-Paired*-*No Sucrose rats slightly reduced their freezing behavior in response to the CS, and no significant difference between phenotypes was detected. The presence of sucrose decreased the freezing behavior in both BER-Paired*-*Sucrose and BEP-Paired*-*Sucrose rats compared with the No Sucrose groups, but only the diminution in BEP-Paired rats was statistically significant. Moreover, the freezing time of BEP-Paired*-*Sucrose rats was significantly different compared with BER-Paired*-*Sucrose rats. Two-way ANOVA revealed the significant effect of the sucrose (*F*_1_,_31_ = 15.170, *p* = 0.001) on the freezing behavior, but not the phenotype (*F*_1_,_31_ = 2.928, *p* = 0.097) or their interactions (*F*_1_,_31_ = 1.429, *p* = 0.241).

### *c-fos* Analyses in Different Brain Regions Involved in Feeding, Stress, and Reward

The *c-fos* mRNA expression was detected by *in situ* hybridization and quantified as the OD. To simplify the presentation, we reduced the number of groups by combining the Control and Tone groups into Non-Paired ones for each phenotype when analyzing the correlation between sucrose intake and *c-fos* mRNA expression.

#### Activation of the Amygdala by the CS Was Not Inhibited by Sucrose Intake

**FIGURE 5:**
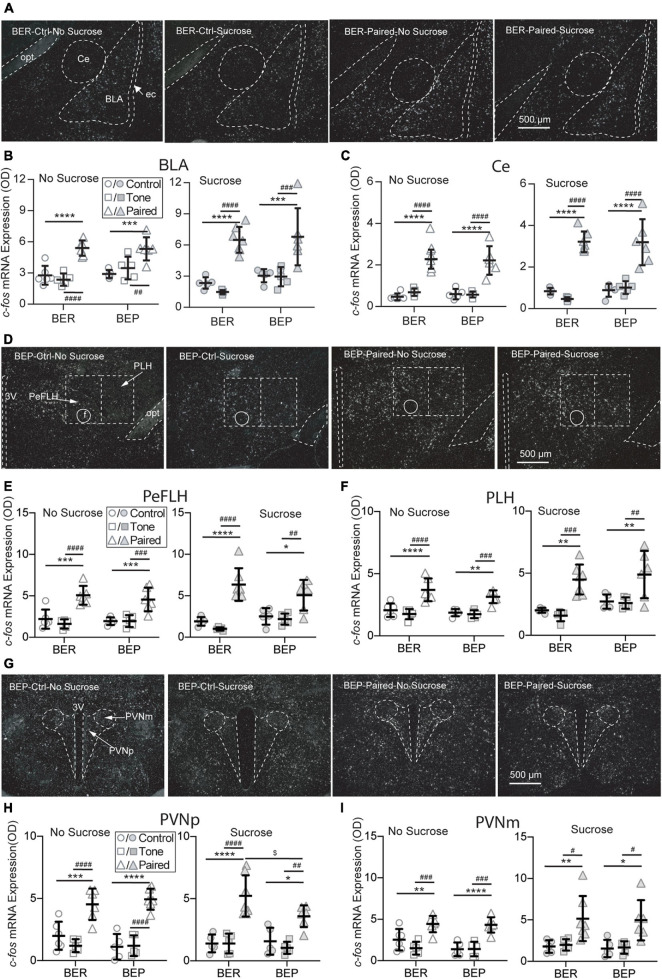
Effects of fear conditioning on the *c-fos* mRNA expression in the amygdala, LHA, and PVN. Representative images of the *c-fos* mRNA *in situ* hybridization signals in the central amygdala (Ce), basal lateral amygdala (BLA), perifornical (PeFLH), and peduncular part (PLH) of the lateral hypothalamus, and parvocellular part (PVNp) and magnocellular part (PVNm) of the paraventricular hypothalamic nucleus marked with broken contours **(A,D,G)**. Relative *c-fos* mRNA expression of No Sucrose and Sucrose groups in the amygdala **(B,C)**, LHA **(E,F)**, and PVN **(H,I)** of BERs and BEPs. 3V, 3rd ventricle; ec, external capsule; f, fornix; opt, the optic tract; rf, rhinal fissure. *Significantly different (*p* < 0.05) from the Control group within the same phenotype. **p < 0.01, ***p < 0.001, ****p < 0.0001. ^#^Significantly different (*p* < 0.05) from the Tone group within the same phenotype. ^##^*p* < 0.01, ^###^*p* < 0.001, ^####^*p* < 0.0001. ^$^Significantly different (*p* < 0.05) from the BER group with the same treatment.

It has long been known that the amygdala plays an important role in the fear conditioning learning and expressing process. The basolateral amygdala (BLA) is the primary site where the association between the CS and US is formed, while the output projections from the central amygdala (Ce) control the freezing behavior and feeding inhibition in response to an aversive CS. Two subregions of the amygdala were analyzed: the Ce and BLA. In both Ce and BLA of both BEPs and BERs, the *c-fos* mRNA expression was more prominent in Paired groups compared with Non-Paired groups, as shown in the representative photos (Figure 5A). Statistical analysis revealed that the Paired groups had higher *c-fos* mRNA expression than the Control and Tone groups in the BLA (Figure 5B) and Ce (Figure 5C) of both phenotypes whether or not there was sucrose access.

#### The Lateral Hypothalamic Area Was Activated by the CS

The LHA is located anterior to the ventral tegmental area (VTA), and posterior to the preoptic area. It integrates information from cortical and subcortical regions, such as the amygdala and basal forebrain networks, and consequently mediates some specific behaviors *via* projecting to downstream circuits involved in reward (e.g., the VTA) and feeding regulation (e.g., brain stem motor pattern generator). Two subregions of the LHA were analyzed: the perifornical part (PeFLH) and the posterior lateral part (PLH). In both subregions of both BEPs and BERs, the *c-fos* mRNA expression was higher in the Paired groups than the Non-Paired groups (Figure 5D), whether or not there was sucrose access. The *c-fos* mRNA levels in the PLH (Figure 5E) and the PLH (Figure 5F) demonstrated a significant increase in the Paired groups compared with Non-Paired groups in both phenotypes, whether or not there was sucrose access in the Test session.

#### The Activation of the Paraventricular Hypothalamic Nucleus by the CS Was Inhibited by Sucrose Intake in the Binge-Like Eating Prone Rats

The PVN is the initiating site of the HPA axis, and neurosecretory neurons in the parvocellular PVN (PVNp) are mainly responsible for the release of corticotropin-releasing factor (CRF) through median eminence in response to stress. The magnocellular component of the PVN (PVNm) releases vasopressin and oxytocin into the systemic circulation upon stress exposure. Two subregions of the PVN were analyzed: the PVNp and PVNm. In both subregions of both BEPs and BERs, the *c-fos* mRNA expression was obviously higher in Paired groups compared with Non-Paired groups, as shown in the representative photos (Figure 5G). Regardless of sucrose access, the *c-fos* mRNA expression in the PVNp (Figure 5H) and the PVNm (Figure 5I) displayed a significant increase in the Paired groups in both phenotypes. With the access to sucrose, the BEP-Paired*-*Sucrose group had lower *c-fos* mRNA expression than the BER-Paired*-*Sucrose group in the PVNp (Figure 5H-right), while sucrose did not significantly change *c-fos* mRNA expression in the PVNm (Figure 5I-right).

#### Persistent Acb Response to Sucrose in the Binge-Like Eating Prone Rats Under Conditioned Fear

**FIGURE 6:**
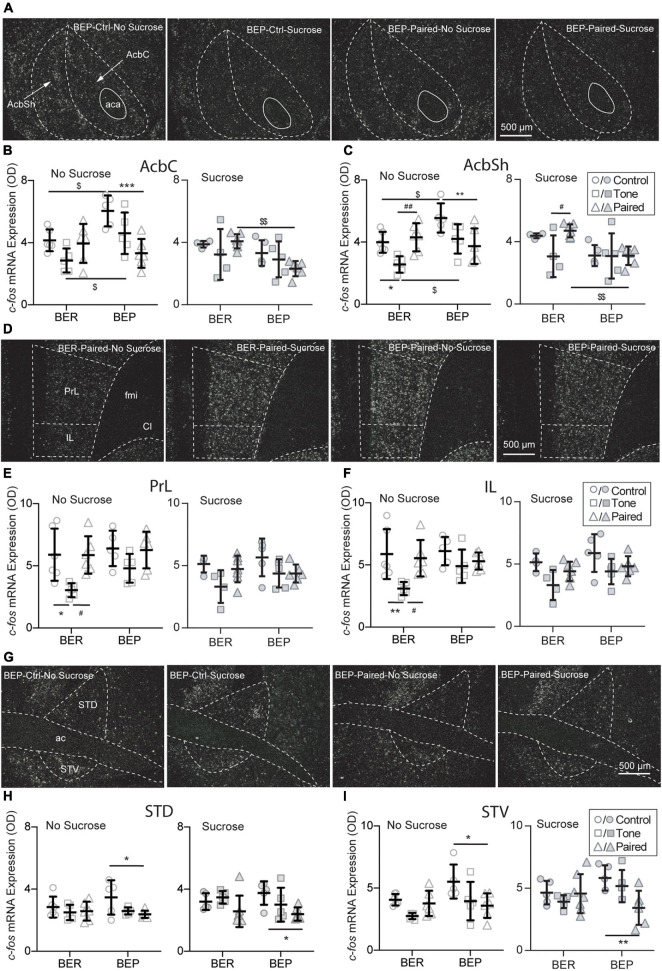
Effects of fear conditioning on the *c-fos* mRNA expression in the Acb, mPFC, and BNST. Representative images of the *c-fos* mRNA *in situ* hybridization signals with the core (AcbC) and shell (AcbSh) part of the nucleus accumbens (Acb), the prelimbic part (PrL), and infralimbic (IL) part of the medial prefrontal cortex (mPFC), and the dorsal part (STD) and ventral (STV) part of the bed nucleus of stria terminalis (BNST) marked with broken contours **(A,D,G)**. Relative *c-fos* mRNA expression of No Sucrose and Sucrose groups and Paired groups in the Acb **(B,C)**, mPFC **(E,F)**, and BNST **(H,I)** of BERs and BEPs. ac, anterior commissure; aca, anterior part of the anterior commissure; LV, lateral ventricle; ic, internal capsule; fmi, forceps minor of the corpus callosum; Cl, claustrum. *Significantly different (*p* < 0.05) from the Control group with the same phenotype. **p < 0.01, ***p < 0.001. ^#^Significantly different (*p* < 0.05) from the Tone group with the same phenotype. ^##^*p* < 0.01. ^$^Significantly different (*p* < 0.05) from the BER group with the same treatment. ^$$^*p* < 0.01.

The dorsal striatum is the central component in the neural circuit of processing the information about the contingencies of the reward stimulus and controlling goal-directed learning process, such as instrumental conditioning. It integrates and processes all reward-related information and subsequently optimizes the reward-related responses. As part of the ventral striatum, the Acb makes the outcome-based predictions. It is responsible for predicting the error-based outcome and constantly updating the predictions about reward and punishment. Two subregions of the Acb were analyzed: the core part (AcbC) and the shell part (AcbSh) of the Acb (Figure 6A). Without sucrose access, the BEP-Non-Paired group had higher *c-fos* mRNA expression in the AcbC and AcbSh than the BER-Non-Paired group. Moreover, the CS pointedly decreased the *c-fos* mRNA expression in the AcbC and AcbSh of the BEP-Paired group compared with the BEP-Control group (AcbC: *p* < 0.001; AcbSh: *p* = 0.007; Figures 6B,C). With the presence of the CS, the BEP*-*Paired*-*Sucrose group had significantly lower *c-fos* mRNA expression in the AcbC and AcbSh than the BER-Paired*-*Sucrose group (Figure 6B-right, Figures 6C-right).

#### The mPFC Was Less Recruited in Response to the CS in Binge-Like Eating Prone Rats With Access to Sucrose

The choice of appropriate defensive behavior (e.g., fight/flight) in a dangerous situation is very important for a better chance of surviving. It has been found that the medial prefrontal cortex is involved in shifting from one strategy to another in various kinds of tasks, including selecting proper defensive responding strategies in stressful situations. Two subregions of the mPFC were analyzed: the prelimbic part (PrL) and the infralimbic part (IL) (Figure 6D). Without access to sucrose, the *c-fos* mRNA expression in both PrL and IL of the BER-Tone*-*No Sucrose group was significantly lower than the BER-Control*-*No Sucrose and BER-Paired*-*No Sucrose groups (Figures 6E,F).

#### The Bed Nucleus of the Stria Terminalis Responded to the CS Only in the Binge-Like Eating Prone Rats

The BNST is composed of a large number of subregions with different functions in stress responding. For example, the anteroventral BNST is highly involved in HPA axis activation. Lesions of this subregion diminish the PVN activation and compress the HPA axis response to restrain the impacts of stress. The anterolateral BNST CRF-expressing neurons project to the PVN, indicating a central modulation action of CRF on the HPA axis. Two subregions of the BNST were analyzed: the dorsal part (STD) and the ventral part (STV) (Figure 6G). The BEP-Paired groups showed significantly lower *c-fos* mRNA expression in the STD (Figure 6H) and STV (Figure 6I) than the BEP-Control groups, regardless of sucrose access.

#### Sucrose Showed Differentiated Effects on the Neural Activities Between Binge-Like Eating Resistant Rats and Binge-Like Eating Prone Rats

**FIGURE 7:**
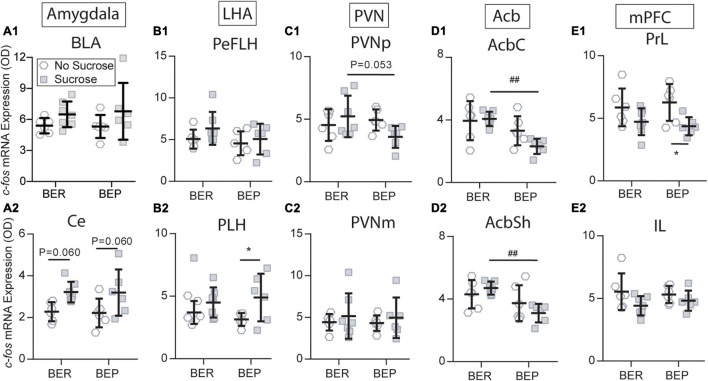
Sucrose changed the *c-fos* expression in response to the fear conditioning in the amygdala **(A1,A2)**, LHA **(B1,B2)**, PVN **(C1,C2)**, Acb **(D1,D2)**, and mPFC **(E1,E2)**. Acb, nucleus accumbens; AcbC, nucleus accumbens core; AcbSh, nucleus accumbens shell; BLA, basolateral amygdala; Ce, central amygdala; IL, infralimbic mPFC; LHA, lateral hypothalamus; mPFC, medial prefrontal cortex; PeFLH, perifornical part of the lateral hypothalamus; PLH, peduncular part of the lateral hypothalamus; PrL, prelimbic mPFC; *significantly different (*p* < 0.05) from the BEP-No Sucrose group. ^##^Significantly different (*p* < 0.01) from the BER-Sucrose group.

Sucrose intake had a tendency to increase *c-fos* mRNA expression in the Ce of both BEPs (*p* = 0.060) and BERs (*p* = 0.060) under conditioned fear (Figure 7A2), but this tendency was not as obvious in the BLA (Figure 7A1) as in the Ce. Therefore, the amygdala only responded to the CS, and it was not influenced by the phenotype or sucrose intake. The LHA has long been known as a feeding regulation center. Under conditioned fear, the BEPs responded to sucrose access with increased *c-fos* mRNA expression in the PLH (*p* = 0.046), which was not found in the BERs (Figure 7B2). This differentiation between BERs and BEPs was not observed in the PeFLH (Figure 7B1). Moreover, sucrose intake decreased the *c-fos* mRNA expression in response to the CS in the BEPs relative to the BERs in the PVNp (Figure 7C1), but not in the PVNm (Figure 7C2).

The BEP*-*Paired group responded to sucrose with significantly lower *c-fos* mRNA expression in the AcbC and AcbSh compared with the BER-Paired group (Figures 7D1, D2). Moreover, sucrose access decreased the *c-fos* mRNA expression in the BEP*-*Paired*-*Sucrose group relative to the BEP-Paired*-*No Sucrose group in the PrL (*p* = 0.028; Figure 7E1), but not in the IL (Figure 7E2). Finally, the sucrose did not change the *c-fos* mRNA expression in the STD and STV of both phenotypes (data not shown).

## Discussion

### Compulsive Eating Was Confirmed in Our Binge-Like Eating Rat Model

In this study, a conflicting situation was created with an aversive CS and palatable food. Consistent with our first hypothesis in the Section “Introduction,” the BEPs showed higher sucrose consumption and lower fear response relative to the BER rats in the presence of the CS during the Test session. However, there actually existed two possible explanations for this result: (1) The higher sucrose intake in BEPs relative to BERs was a result of lower sensitivity to the CS in BEPs. (2) The BEPs had higher motivation for sucrose than the BERs, which overcame the fear for the CS and sucrose had a stronger anxiolytic effect on the BEPs relative to the BERs, which diminished their fear response to the CS. The second explanation was exactly our hypothesis. To confirm our hypothesis, we had to exclude the possibility of the first explanation. To solve this problem, we set the No Sucrose groups as a control. We can see that the freezing behavior was comparable between BEPs and BERs when they had no access to sucrose during both Fear Conditioning training sessions and the Test session. This finding perfectly confirmed that BERs and BEPs had comparable sensitivity to the CS and that the attenuated effects of the CS were indeed a result of abnormally high motivation for sucrose in the BEPs. Combined with the decreased freezing behavior in BEP-Paired-Sucrose rats, it also indicated a stronger anxiolytic effect of sucrose on the BEPs relative to the BERs.

Until now, we have observed all aspects of compulsive eating in the BEPs in our BED rat model: habitual overeating in the Appetitive sessions, as well as overeating to relieve negative sensation and overeating despite aversive consequences in the Test session. In an acute stressful environment, the animals have to recruit their energy and attention for a swift and proper response, and concurrently inhibit other housekeeping activities such as food intake, digestion, and reproduction. In the BERs, the conditioned fear inhibited the licking behavior not only during tones but also between tones, a relatively less stressful but uncertain situation. In the BEPs, the licking behavior was not affected between tones, suggesting a deficiency of devaluating the palatable food when confronted with potential dangers. Similarly, both humans and animals with eating disorders appear to have some struggles in suppressing food-seeking and -taking in an emergent situation (Oswald et al., 2011). Overeating of palatable food despite aversive consequence happens when an abnormally high motivation for palatable food inhibits the normal function of the stress-responding system (Voon, 2015).

### Exploration of Neural Mechanisms Underlying the Compulsive Eating

To understand the neural mechanisms underlying the different feeding and freezing behavior under conditioned fear, we analyzed the *c-fos* mRNA expression in brain regions involved in stress responding, feeding regulation, and reward processing. In Figures 5, 6, we tested two factors, phenotype and fear conditioning, to see whether the analyzed brain regions of BER and BEP respond differently to fear conditioning when they had no access to sucrose, or when they had access to sucrose. In this way, we can know if there is a difference between BER and BEP rats in their brain activities in response to fear conditioning, without considering the effect of sucrose access. In Figure 7, we put together all four Paired groups and tested two other factors, phenotype and sucrose, to see if sucrose can change the brain activities in response to fear conditioning and if this effect is different between BER and BEP rats. Consistent with our second hypothesis in the Section “Introduction,” we found different neuronal activities in many brain regions between BERs and BEPs that could be underlying their different sucrose consumption and freezing behavior under conditioned fear.

#### The Acb, Medial Prefrontal Cortex, and Lateral Hypothalamic Area Were Possibly Underlying the Abnormally High Motivation for Sucrose in the Binge-Like Eating Prone Rats

Higher tonic Acb activity might be underlying the habitual overeating of the BEPs. Distinct cell types and input projections in the Acb are in charge depending on the availability of palatable food, representing productive and unproductive rewarding seeking (Lafferty et al., 2020). A popular hypothesis is that the hyperpolarization of medial spinal neurons (MSNs) in the Acb is primarily underlying the appetitive motivation (wanting), and inhibition of these GABAergic neurons can disinhibit the downstream targets, such as the ventral pallidum, VTA, and LHA, and promote hedonic responses (liking) and continuation of feeding behaviors (Castro et al., 2015). Extended access to palatable food can induce hyperphagia and compulsive eating in rats, accompanied by gradually worsened responsiveness of reward system, decreased baseline extracellular dopamine in the Acb (van de Giessen et al., 2014), and downregulated striatal dopamine D2 receptors (D2Rs) (Johnson and Kenny, 2010). Considering the inhibitory function of the D2Rs, these changes could decrease the tonic inhibition of the Acb, and possibly contributed to the higher baseline Acb activity in the BEPs.

Consistently, without the stimulation of CS and sucrose, the BEPs showed a higher baseline activity in the Acb compared with the BERs, indicating a stronger motivation for palatable food in an environment where sucrose was usually available. Thus, we propose that tonic reward response was diminished in the BEPs, and consequently, they had to eat more and faster to activate the reward system. It could drive a higher motivation and expectation for sucrose in the BEPs, which could explain the shorter first lick latency of the BEPs when they got access to sucrose. We hypothesize that the hyperactivity of the Acb of BEPs in “resting state” reflected higher motivation (wanting) for sucrose when it is unavailable (unproductive seeking), while the larger amplitude of decrease of the Acb activation might exaggerate hedonic rewarding value of sucrose (liking) in BEPs.

The persistent hedonic response in the Acb and diminished recruitment of the mPFC in the presence of palatable food in the BEPs were possibly underlying their deficient devaluation of palatable food in face of aversive consequences. It is also well known that inhibition of the Acb increased, and stimulation decreased, intake of food (Chometton et al., 2020; Yang, 2021). Under conditioned fear, the sucrose failed to significantly inhibit the activity of the Acb in BERs. It indicates that the dynamic function of the Acb depends on the current salience of stimuli and that the hedonic value of sucrose for BER decreased in the face of the CS. We can comprehend this disrupted hedonic response as an evidence of devaluation of palatable food in the presence of aversive stimuli. Increased activity of the projection from the AcbSh D1R (dopamine type 1 receptor expressing) MSNs to the LHA GABA neurons has been proven underlying the inhibition of feeding behavior by salient external stimuli (O’Connor et al., 2015). On the contrary, the CS did not change the pattern of responding to sucrose in the BEPs, suggesting a resistance to the devaluation of palatable food by potential aversive consequences.

Considering its reciprocal connections with the amygdala and dense projections to the LHA and Acb, the mPFC may also play an important role in devaluating the palatable food under a stressful situation, and modulating value-based decision-making. A recent study (Christoffel et al., 2021) found that anterior paraventricular thalamus (aPVT) and mPFC projections to the Acb differentially regulate the rewarding properties of high-fat food. Inhibition of the glutamatergic mPFC-Acb projection promotes the acquisition of binge eating on high-fat food, while stimulating the same project would suppress the hedonic feeding. Consistently, the BEPs in this study displayed inhibited PrL activity with access to sucrose under conditioned fear, but not the BERs. This decreased PrL activity in the BEPs was very likely responsible for their deficient decision-making adjustment and persistent sucrose intake under the conditioned fear.

#### The PVNp Was Likely an Important Target of Sucrose for Its Stronger Anxiolytic Effects on the Binge-Like Eating Prone Rats

The amygdala and BNST were not likely the target of sucrose for its anxiolytic effects. For many years, the amygdala has been considered as the emotional center, especially for coding the conditioned fear response. Consistent with the functions of the BLA and Ce in the fear conditioning acquisition and expression, respectively, the CS activated both of them without any difference between the BERs and BEPs. This is consistent with the comparable fear response of the BEPs and BERs in Fear Conditioning sessions and the Test session when they had no access to sucrose. Petrovich previously showed that lesions of the Ce, not the BLA, abolished the feeding-inhibiting effect of an aversive CS (Petrovich et al., 2009). Consistent with this special function of the Ce, this study found that the Ce further increased its activity in response to sucrose access in the presence of an aversive cue, compared with the cue itself. It indicated the involvement of the Ce in the devaluation of palatable food in face of potential aversive consequences. Surprisingly, compared with Paired-No Sucrose groups, the activities of the BLA and Ce in Paired-Sucrose groups of BER and BEP rats were not inhibited by sucrose intake in this study, suggesting that the amygdala is not likely the functioning site of the anxiolytic effects of palatable foods, or at least indicating that we need to look into the activities of subtypes of neurons for the underlying mechanisms.

The BNST is part of the “extended amygdala,” along with the Ce and caudal Acb. Substantial evidence supports the involvement of the BNST in the fear and anxiety response to conditioned and unconditioned stimuli. The BNST controls the stress-induced seeking and consumption of drugs and palatable food by receiving stress information from the Ce (Erb et al., 2001) and projecting to the VTA (Kudo et al., 2012). In human patients, the severe obsessive–compulsive disorder could be alleviated by electrical deep brain stimulation in the BNST (Luyten et al., 2016).

In this study, sucrose intake inhibited the *c-fos* mRNA expression in the Acb of the BEPs, but not in the BERs. The BEPs showed decreased BNST activity in response to the CS, but not BERs. Lesions of the BNST did not affect the fear response to an aversively conditioned sound, but decreased the response to another unconditioned sound, indicating a fear-generalization function of the BNST (Duvarci et al., 2009). Considering this discrimination inhibitory effect of the BNST, its lower activity in the BEPs during the Test session might explain the unaffected licking behavior of BEP-Paired rats between tones compared with BER-Paired rats. This different BNST response to the CS between the BEPs and the BERs was observed even without sucrose access, suggesting different stress responding strategies between phenotypes, which might be involved in the development of stress-induced overeating in BEPs. Anyway, the sucrose access failed to induce significant change in the activity of the BNST in BERs and BEPs under conditioned fear, excluding it as a direct functioning site of sucrose for an anxiolytic effect.

The enhanced anxiolytic effect of sucrose on the BEPs was regulated by diminished PVNp response to the CS by sucrose access. As part of the HPA axis, the neuroendocrine neurons of the PVN are responsible for the secretion of CRF and vasopressin in response to stress stimuli. Not surprisingly, in Paired groups, the CS significantly upregulated the *c-fos* mRNA expression in the PVN of both BEPs and BERs, which is consistent with many previous IEG-based (immediate early gene-based) mapping studies (Honkaniemi et al., 1994). It is also known that palatable food, such as sucrose and lard, could attenuate the stimulating effect of stress on the HPA axis (Foster et al., 2008). In this study, sucrose ingestion attenuated the PVNp response to stress in the BEPs compared with the BERs. This result is consistent with the freezing behavior. However, similar to the amygdala, there is no evidence showing that the PVNp directly controls the freezing behavior. Nevertheless, we can still hypothesize that sucrose has a stronger anxiolytic effect on the BEPs than the BERs, which can also explain the higher sucrose intake of the BEPs during the phenotype classification. Decreased HPA axis response to stress in the BEPs has been observed in another study with the BED rat model, demonstrating as attenuated plasm corticosterone, and CRF mRNA expression in the PVN in response to the foot shock stress (Calvez et al., 2016). The parvocellular part of the PVN sends glutamatergic projections to the Acb, and pharmacogenetic stimulation of the PVN–Acb projections can decrease the intake of highly palatable food (Smith et al., 2020). These findings are consistent with results in this study. In conclusion, the hedonic value of palatable food has strong anxiolytic effect *via* inhibiting the PVN activity; conversely, the mental status regulates the hedonic value of palatable food *via* the PVN–Acb pathway.

#### The Feeding Center Lateral Hypothalamic Area Was Possibly an Integrating Site of Rewarding and Stress Responding Information to Regulate the Compulsive Eating in the Binge-Like Eating Prone Rats

Studies with the retrograde tracing technique revealed that the BLA and Ce send projections to the ventral and dorsal LHA, respectively (Reppucci and Petrovich, 2016). Noxious stimulation increases *c-fos* expression in the LHA (Bullitt, 1990), and lesions of the LHA significantly decrease arterial pressure response to a CS (LeDoux et al., 1988). The activation of the LHA by CS in this study was likely induced by activating projections from the amygdala and might play a role in the devaluation of sucrose by projecting to the ventral tegmental area (VTA) (Nieh et al., 2015, 2016). Moreover, the LHA is recognized as the feeding center, and it receives the leptin information from the arcuate nucleus (ARC) and changes the hedonic value of the nutrition, which is important for the regulation of homeostatic feeding, *via* projections to the reward areas, such as the VTA (Domingos et al., 2013). The post-ingestive rewarding effect of sucrose might be the response for the increase of PLH activity of the BEPs under conditioned fear that likely contributed to the inhibiting effect of sucrose on the freezing behavior. Thus, we conclude that the CS also had a strong impact on the activity of the LHA, and under the conditioned fear, the abnormally higher PLH activity in the BEPs finally drove their compulsive eating.

In conclusion, the response of Acb and mPFC to the palatable food is dynamically modulated by the current value of the food and that the Acb and mPFC participate in coding for the devaluation of palatable food in the face of potential aversive consequences. The compulsive eating observed in the BEPs was likely facilitated by deficits in devaluating the rewarding effects of sucrose, represented by attenuated recruitment of the mPFC, persistent Acb response to sucrose intake, and increased LHA activity in stressful situations. The interaction between the rewarding system and the stress responding system facilitates the development of the BEP phenotype, leading to abnormally high motivation for binging on high-sugar and high-fat diet over healthier food. When the hedonic system hijacks the homeostatic system and the stress-responding system, compulsive eating will attenuate the normal response to potential aversive consequences.

### Drawbacks of This Study

This study used female rats, rather than male rats, because the prevalence of the BED is higher in women compared to men (Cossrow et al., 2016). However, this choice leads to an inevitable problem—the estrus cycle and hormone fluctuations in female animals. The fear response and food intake of the female rats in this study might have been influenced by the estrous phase of individual animals in the Test session. Unfortunately, the estrous phase was not taken into consideration in this study, because excluding some animals from each group, especially the control groups, or separating them into subgroups according to their estrous phases would make the sample size too small for statistical analysis. Future studies with a larger number of animals are necessary to solve this problem.

### Fear Conditioning Test as a Good Paradigm for Analyzing Compulsive Eating

To our knowledge, this is the first study that combined the fear conditioning and binge eating animal model to observe the impulsivity of binge eating, and explored the neuronal activities underlying the regulation of feeding behavior, fear response, and compulsive eating under a stressful situation. The fear conditioning paradigm has many advantages over direct foot shock. For example, if we want to do some electrophysiological recording during the aversive stimulus, the foot shock will generate noise in the signals, and even damage the amplifiers, which can be avoided with the conditioned cues, such as light and sound. Our findings indicate some potential targets for the treatment of the BED. For example, we can suppress the craving for palatable food by decreasing the baseline activity of the Acb and enhance the devaluation of palatable food by increasing the recruitment of the mPFC, and modifying the Acb response to palatable food. The conflicting test with fear conditioning paradigm developed in this study can provide a useful tool to explore the brain and endocrine mechanisms of the occurrence of the BED, and may provide a platform to test and compare the pharmacotherapies to suppress the compulsive eating of palatable foods.

## Data Availability Statement

The original contributions presented in the study are included in the article/supplementary material, further inquiries can be directed to the corresponding authors.

## Ethics Statement

The animal study was reviewed and approved by the institutional Université Laval Animal Care Committee, Laval University, Quebec, Canada. The ethics number is 2017-013-1.

## Author Contributions

All authors listed have made a substantial, direct, and intellectual contribution to the work, and approved it for publication.

## Conflict of Interest

The authors declare that the research was conducted in the absence of any commercial or financial relationships that could be construed as a potential conflict of interest.

## Publisher’s Note

All claims expressed in this article are solely those of the authors and do not necessarily represent those of their affiliated organizations, or those of the publisher, the editors and the reviewers. Any product that may be evaluated in this article, or claim that may be made by its manufacturer, is not guaranteed or endorsed by the publisher.
